# App-based daily self-measurement of impedance in cochlear implant users

**DOI:** 10.3389/fneur.2025.1618031

**Published:** 2025-07-04

**Authors:** Sarah Vormelcher, Cornelia Batsoulis, Daniel Kley, Michael Mair, Andreas Büchner

**Affiliations:** ^1^Department of Otorhinolaryngology, Hannover Medical School, Hannover, Germany; ^2^MED-EL Research Center, MED-EL Medical Electronics GmbH, Hannover, Germany; ^3^MED-EL R&D, MED-EL Medical Electronics GmbH, Innsbruck, Austria; ^4^Department of Otorhinolaryngology and Cluster of Excellence “Hearing4all”, Hannover Medical School, Hannover, Germany

**Keywords:** cochlear implant, daily impedance measurement, electrode impedance, electrical stimulation, remote impedance monitoring, impedance fluctuation, app usability

## Abstract

**Introduction:**

Impedance telemetry measurements in cochlear implant (CI) recipients are commonly used to assess CI electrode functionality and provide valuable insights into inner ear conditions. However, these measurements usually take place only during surgery and at clinical follow-up appointments, offering limited temporal resolution of impedance changes. This study aimed to address this gap by implementing daily impedance monitoring using a smartphone app.

**Methods:**

A prospective study evaluated the usability of a research app for remote impedance measurements over 4 months following standard CI surgery with a MED-EL FLEX28 electrode. Impedance was recorded twice daily (morning and evening). The mean impedance across all electrode channels was analyzed for four postoperative time periods: early postoperative (up to day 10 postoperatively), late postoperative (from day 11 to ~4 weeks), intensive fitting (from ~4 weeks postoperatively to ~7 weeks) and regular hearing phase (from ~7 weeks to 4 months). Two CI fitting approaches were compared: activation during the early postoperative phase (early activation, EA) and activation during the intensive fitting phase (conventional activation, CA). Morning-to-evening differences in impedance (MED) were also examined.

**Results:**

The app demonstrated an overall usage rate of 66% (*n* = 28), indicating moderate-to-high adherence. Except for higher evening impedance values with CA in the late postoperative phase, no significant differences in mean impedance between the fitting approaches were observed (EA: 6.46 kΩ, *n* = 11; CA: 7.82 kΩ, *n* = 11; *p* = 0.04). Significant differences in MED were found during the early postoperative phase (EA: 0.06 kΩ, *n* = 8; CA: −0.18 kΩ, *n* = 10; *p* = 0.04) and the late postoperative phases (EA: 0.85 kΩ, *n* = 11; CA: 0.03 kΩ, *n* = 11; *p* < 0.001).

**Conclusion:**

Remote impedance measurements via the app can be made over an extended postoperative period. The increased measurement frequency allowed for detailed characterization of impedance dynamics, particularly around the onset of electrical stimulation. No clinically relevant difference in mean impedance was found between EA and CA groups. Daily fluctuations showed consistently lower evening values after stimulation onset. These findings highlight the potential value of this approach for enhancing postoperative CI management.

## Introduction

In recent years, even individuals with moderate hearing loss have become candidates for cochlear implantation (CI). This shift underscores the importance of both structural and functional hearing preservation, which are known to improve CI outcomes (1–3). Impedance measurements are commonly used to assess CI electrode functionality, but have also been explored as a potential indicator of inner ear conditions such as intracochlear inflammation (4–6). Since inflammation may compromise residual acoustic hearing, early detection through impedance monitoring could help identify users at risk of loss of residual hearing. While inflammation-related changes can occur at any time after implantation, even years later, they remain relatively rare (5, 7).

Postoperatively, a transient increase in impedance is typically observed as a response to electrode insertion trauma (8), primarily due to protein adsorption on the electrode surface and to modifications at the electrode-electrolyte interface (9, 10). *In vitro* studies suggest that this protein deposition is reversible (11, 12), as impedance decreases following the onset of electrical stimulation and subsequently stabilizes (13–15). However, long-term biological processes such as cellular proliferation and tissue encapsulation (fibrosis) around the electrode lead to a gradual and often irreversible increase in impedance. Several studies have confirmed these findings, particularly as advancements in impedance analysis now allow differentiation of its individual subcomponents in humans (9, 13, 16–20).

To counteract foreign body responses and reduce electrode impedance, pharmacological interventions such as intracochlear administration of steroids like dexamethasone or triamcinolone have been investigated. These have shown to be effective (20–24).

While routine clinical impedance measurements provide valuable physiological insights, they are typically performed at discrete follow-up appointments and as such offer only limited temporal resolution. Impedance values tend to stabilize ~1-year postimplantation, although reported stabilization times vary widely, from weeks to years (25–29). At our center, CI recipients are typically discharged 1–3 days postsurgery and return for their first follow-up visit after ~4 weeks (30, 31), with no impedance measurements recorded in the interim.

Although self-impedance measurements have already been made possible by previous technical developments of the different CI manufacturers, the novelty of this study lies in the frequency and temporal resolution of the measurements. Few studies have investigated long-term impedance behavior in CI recipients, and only two have systematically examined daily impedance fluctuations, one of which restricted measurement to the early postoperative period, while the other analyzed impedance trends in experienced CI users (27, 32). As a result, the precise physiological mechanisms underlying impedance evolution in the months and years following CI surgery remain poorly understood. In particular, the impact of the initiation of electrical stimulation on impedance progression has yet to be systematically examined (11, 14, 15, 30, 31, 33).

This study aimed to address this gap by implementing daily self-monitoring of impedance levels using a smartphone app for several months postsurgery. The increased measurement frequency compared to clinical visits aimed to provide a more detailed understanding of impedance behavior and facilitate early recognition of data patterns. We investigated the app's usability for conducting daily impedance measurements and separated participants into two groups based on their activation strategy. The first group comprised those with early activation (EA), who underwent their first electrical stimulation within 1–3 days postoperatively and were encouraged to use their audio processor daily. The second group comprised those with conventional activation (CA), who did not receive audio processor activation until the intensive fitting week (~4 weeks postoperative) and were encouraged to use their processor daily thereafter. By comparing the impedance trends in these groups over a period of 4 months, we sought to provide more detailed insight into the temporal dynamics of the physiological processes that occur within the cochlea after implantation and to investigate the influence of electrical stimulation.

## Materials and methods

### Ethics and informed consent

The study was conducted in accordance with the World Medical Association's Declaration of Helsinki and was approved by the local ethics committee at Hannover Medical School (reference numbers 9115_BO_S_2020 and 9115_BO_S_2022).

All participants were informed of the study procedures and provided their explicit informed consent before the start of the study.

### Study design, inclusion criteria, and participant details

This was a prospective, monocentric interventional study conducted at the German Hearing Center of the Hannover Medical School. The study began in June 2020, and the last enrolled participant reached their 4-month postoperative follow-up in August 2023.

Participants had to meet the following inclusion criteria:

- Age ≥18 years
- Normal inner ear anatomy
- First cochlear implantation in the ear to be measured
- Use of a MED-EL SONNET2 audio processor
- Willingness to use a smartphone
- Residency in Germany
- Proficiency in German to understand study procedures and provide informed consent

A total of 28 participants (21 male and 7 female) were included in the study. Demographic data for each participant are shown in Supplementary Table S1. Their mean age at implantation was 58.3 ± 15.7 years and the mean unaided preoperative hearing level at 500 Hz was 80.9 ± 15.4 dB HL. All had undergone a standard CI implantation procedure with a round window insertion. All received a SYNCHRONY or SYNCHRONY 2 implant with a FLEX28 electrode array (MED-EL, Innsbruck, Austria). Measurements were taken from 17 right ears and 11 left ears.

### Telemetry App

The research software Telemetry App enabled participants to perform self-measurements of electrode impedance after pairing their CI audio processor with the app. The impedance measurement was manually initiated by the participant by navigating through four levels within the Telemetry App. No push notifications or automated reminders were implemented within the app. To ensure full functionality and comparability across participants, the Telemetry App was specifically developed and tested for the Android mobile operating system and deployed on a standardized smartphone model (Samsung Galaxy A10) with internet access, which was provided to all participants during the initial postoperative implant check, typically 1–3 days after surgery.

### Study procedure

In accordance with the clinical procedures at the Hannover Medical School, all participants underwent functionality checks of the implant and measurement of impedance field telemetry (IFT) using the MAESTRO software (MED-EL, Innsbruck, Austria). This was performed during surgery, and again at the postoperative implant check which typically took place 1–3 days after surgery before discharge from the hospital.

#### Activation strategy

**Figure 1:**
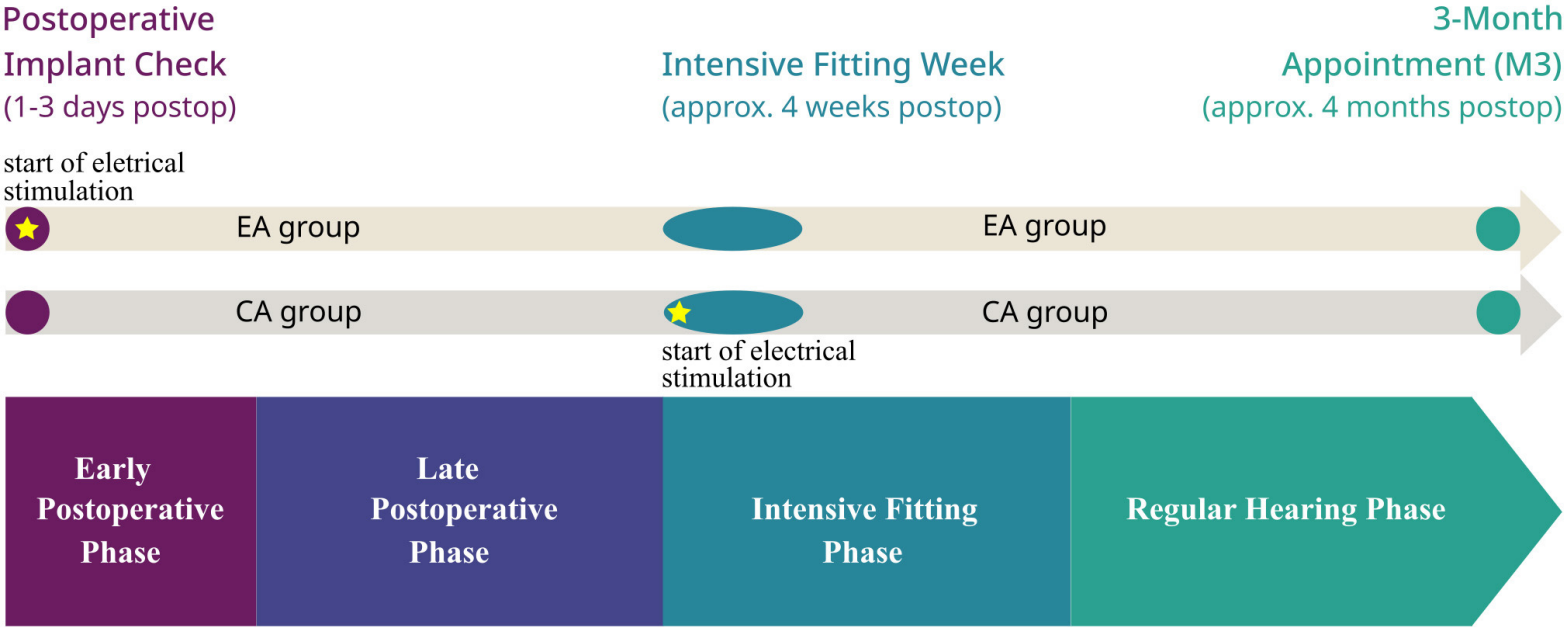
Timeline after CI surgery for the early activation (EA) and conventional activation (CA) groups. Circles and ovals depict clinical appointments, boxes depict the defined postoperative phases, and stars depict the start of electrical stimulation.

Following surgery, participants received one of two activation strategies, as determined by routine clinical practice (Figure 1). Those in the EA group received a preliminary fitting map with electrical stimulation of the implant at the time of the postoperative implant check, meaning that the first electrical stimulation was applied 1–3 days after surgery. Participants were instructed to use the processor in their daily lives. For those in the CA group, no electrical stimulation was applied until the intensive fitting week, which took place ~4 weeks after surgery. To enable the wireless connection to facilitate impedance measurements with the Telemetry App, a “zero map” (with all stimulation levels set to zero) was applied to the audio processor.

#### Telemetry App participant instruction

All participants were trained by the study team on how to use the Telemetry App. The first impedance measurement was performed immediately after the postoperative implant check in the clinic, once the audio processor was connected to the app.

After the initial supervised measurement, participants were instructed to perform impedance measurements by themselves at home, measuring all 12 electrode contacts twice a day—once in the morning immediately after attaching the processor, and again in the evening just before removing it. This measurement routine was to be maintained for a period of 4 months following implantation.

#### Follow-up procedures

All participants returned to the clinic for their routine follow-up appointments. These included the intensive fitting week, which took place ~4 weeks after surgery, and the 3-month follow-up appointment (M3), which was scheduled for ~4 months postimplantation (Figure 1).

The fitting procedure for determining the most comfortable levels (MCLs) and thresholds (THRs) was comparable for both groups, although the timing differed. As the initial implant check in the EA group, conducted 1–3 days postoperatively, mirrored the first-day fitting procedure of the CA group during the intensive fitting week, the EA group effectively received one additional day of overall fitting time. Regardless of the initiation of electrical stimulation, both groups received fine-tuning of the MCL and THR values during the intensive fitting week.

### Usage rate of the Telemetry App

To assess how frequently the Telemetry App was used, we calculated the rate of daily app usage. A day was considered active for this analysis if at least one impedance measurement was recorded.

For each participant, the total number of days between the first measurement (postoperative implant check) and the last study visit (M3 follow-up appointment) was calculated. Similarly, the number of active days was determined. The usage rate was then calculated as the ratio of these two.

Participants who completed fewer than one-third of the total possible measurement days were excluded from the analysis to ensure data quality and maintain the analytical validity of the longitudinal impedance assessment.

### Impedance measurement parameters

Compared to the clinical MAESTRO software (version 8 and 9), which used a phase duration of 24 μs with a stimulation amplitude of 302 μA, the Telemetry App used a phase duration of 23 μs and a stimulation amplitude of 150 μA. In both cases, voltage measurements were taken at the end of the second phase of a biphasic pulse, and impedance values were calculated by dividing the measured voltage by the applied stimulation current. The analysis focused on classical “clinical” impedance values (corresponding to the diagonal elements of the impedance matrix). A distinction between individual subcomponents, such as access or polarization impedance, was not made. Stimulation parameters during regular implant use are variable and defined by the patient's clinical fitting map.

### Postprocessing

The impedance values and corresponding timestamps from the Telemetry App were transmitted via a secure internet connection to a SQL cloud database in pseudonymized form. Data was regularly exported from this database to an Excel file and subsequently analyzed using Python version 3.12.7 with the libraries pandas, numpy, matplotlib, seaborn, scipy, and statannotations.

### Time allocation of app measurements

Measurements were categorized as either morning or evening measurements. Morning measurements took place between 04:00:00 a.m., and 12:59:59 p.m. Evening measurements took place between 04:00:00 p.m., and 01:59:59 a.m.

Measurements that did not fall into either category were excluded from the analysis. If a participant performed multiple measurements within the same time period on a given day, the first measurement was retained for the morning category and the last retained for the evening category. Since the first measurement with the Telemetry App was conducted during the postoperative implant check in the clinic, it was classified as a morning measurement regardless of the actual time of day.

The observation period was divided into several postoperative phases (Figure 1):

*Early postoperative* (up to day 10 postoperative). During this phase, the EA group had already started receiving electrical stimulation through a preliminary fitting, while the CA group had not.

*Late postoperative* (from day 11 to 1 day before the start of the intensive fitting week). During this period, the EA group continued receiving electrical stimulation while the CA group still had not.

*Intensive fitting* (from the beginning of the intensive fitting week up to 21 days after). This phase represents the period when both groups received fine-tuning of the electrical stimulation during the intensive fitting week. To ensure that electrical stimulation was fully established, only measurements from the second day of the fitting week were considered for analysis in this phase, as in the CA group no electrical stimulation occurred in the morning of the first day, since fitting took place throughout the day.

*Regular hearing* (from 22 days after the beginning of the intensive fitting week up to 4 months postoperative). During this phase, both groups had fully adapted to electrical stimulation.

The first day of the intensive fitting week was used as the reference point (*t*_0_). To represent measurements before *t*_0_, data were depicted up to −30 days, defined by the cohort's minimum number of days between the postoperative implant check and the intensive fitting week. The endpoint of the observation period was determined by the cohort's maximum recorded number of days, up to 4 months postoperatively.

### Impedance measurements across time in the app cohort

To ensure that the impedance analysis was based on valid impedance values, channels close to supply voltage saturation or disabled channels were excluded from the analysis and treated as missing values. The averaged impedance across all non-excluded channels (C1–C12) was analyzed over time for both morning and evening measurements separately for EA and CA groups.

### Morning-to-evening differences in app impedance

In addition to analyzing the absolute impedance values measured with the Telemetry App, the within-participant morning-to-evening impedance differences (MED) were calculated for the four postoperative phases in both the EA and CA groups of the app cohort. To calculate the MED of all non-excluded channels (C1–C12), the evening impedance values were subtracted from the corresponding morning values for each day.

### MAESTRO impedance data comparison: app vs. routine cohort

Due to the relatively small cohort of app users and the presence of partially incomplete measurements, a larger comparison group (the “routine cohort”) was constructed using retrospective clinical IFT data derived from routine appointments of CI users from the German Hearing Center. All were adults who had received a SYNCHRONY or SYNCHRONY 2 implant with a FLEX28 electrode array as their first implantation during the same implantation period as the app cohort. For comparison, IFT values from the app cohort were used; these values were recorded using the clinical MAESTRO software during their regular follow-up appointments, rather than through the app itself.

Users in the routine cohort received one of two activation strategies, as determined by routine clinical practice and were also stratified into EA and CA groups based on their activation strategy. IFT data were collected using MAESTRO (version 8 and 9). The clinical timepoints available for this group were intraoperative (intraop), at the end of the intensive fitting week (final fitting), and at their 3-month follow-up (M3).

As with the app cohort, channels close to supply voltage saturation or disabled channels were excluded from the analysis and treated as missing values.

### Statistical analysis

To identify outliers in the averaged impedance values across all non-excluded channels, the interquartile range (IQR) method was applied separately for EA and CA groups across the different postoperative phases or clinical timepoints. Values beyond three times the IQR below the first quartile or above the third quartile were defined as extreme outliers.

Extreme outliers were removed from analysis to account for individual participant effects that may have caused abnormally elevated impedance values. Based on clinical experience, these effects are often due to air bubbles at the electrode array (particularly in the early postoperative phase), swelling at the incision site, or thicker skin flaps.

#### Impedance measurements across time in the app cohort

For the comparisons between EA and CA groups regarding app impedance measurements across time, we consistently compared the averaged impedance values across all non-excluded channels between the two groups. Normality of the data was assessed within each postoperative phase (morning and evening separately) using the Shapiro–Wilk test. If the data for a given phase were not normally distributed, the Mann–Whitney *U*-test was applied. Otherwise, Welch's *t*-test was used to account for potential inequality of variances. These procedures were applied to all impedance comparisons throughout the different postoperative phases.

#### Morning-to-evening differences in app impedance

For comparisons between EA and CA groups in the MED analyses, the averaged impedance values were tested for normality within each postoperative phase using the Shapiro–Wilk test. If the data were not normally distributed, the Mann–Whitney *U*-test was applied. Otherwise, Welch's *t*-test was used to account for potential variance inequality between the independent groups.

#### MAESTRO impedance data comparison: app vs. routine cohort

For comparisons of the MAESTRO measurements between the app cohort and the clinical routine cohort, the averaged IFT values across all non-excluded channels (C1–C12) were analyzed separately for each clinical timepoint. Normality was assessed using the Shapiro-Wilk test within each group. Depending on the distribution, either the Mann–Whitney *U*-test or Welch's *t*-test was used to compare the two independent cohorts.

The significance threshold was α = 0.05. To adjust for multiple comparisons, Bonferroni correction was applied, adjusting for the number of comparisons performed in each analysis.

## Results

### Usage rate of the Telemetry App

**Figure 2:**
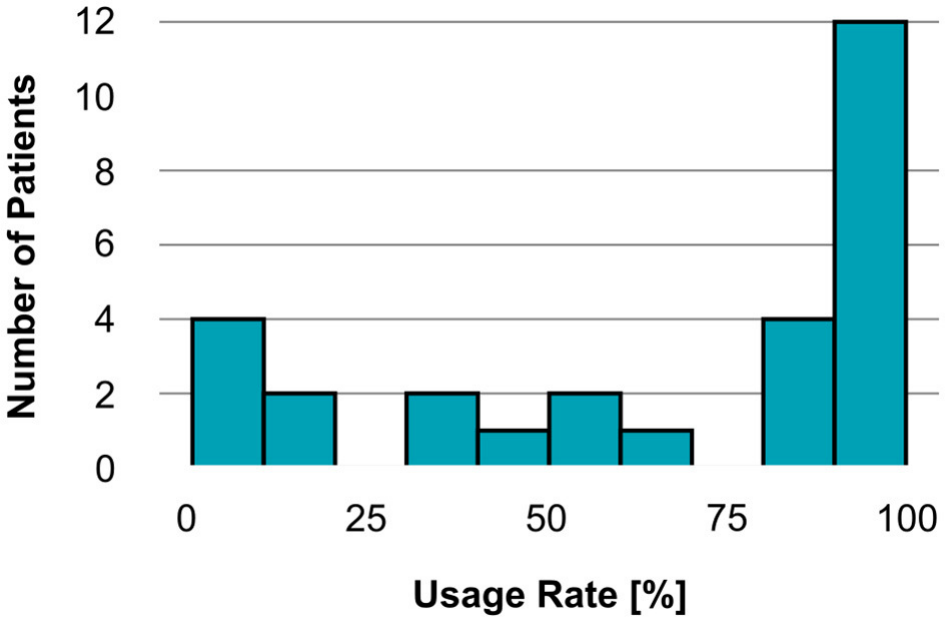
Usage rates of the Telemetry App (*n*= 28), showing the number of participants across usage rates (%), calculated as (active days/total possible days) × 100 for the period between clinic appointments (postoperative implant check to M3).

For the observation period of the app cohort (between the postoperative implant check and M3), a total of 3,867 possible measurement days were recorded across all 28 participants. Within this period, usable (i.e., non-excluded) impedance measurements were obtained on 2,547 days, corresponding to an overall usage rate of 65.9% across all participants, indicating a moderate to high adherence to the study protocol. Despite this, some participants had substantially lower individual usage rates. Figure 2 shows the distribution of individual usage rates.

To ensure a reliable impedance analysis over time, *n* = 6 participants with <33.33% of days with impedance measurements were excluded from the impedance analysis. Supplementary Figure S1 presents histograms illustrating the distribution of measurement time points for the analyzed morning and evening categories. Morning measurements most frequently took place between 08:00 and 10:00, while evening measurements most frequently took place between 18:00 and 20:00.

### Impedance measurements across time in the app cohort

**Figure 3:**
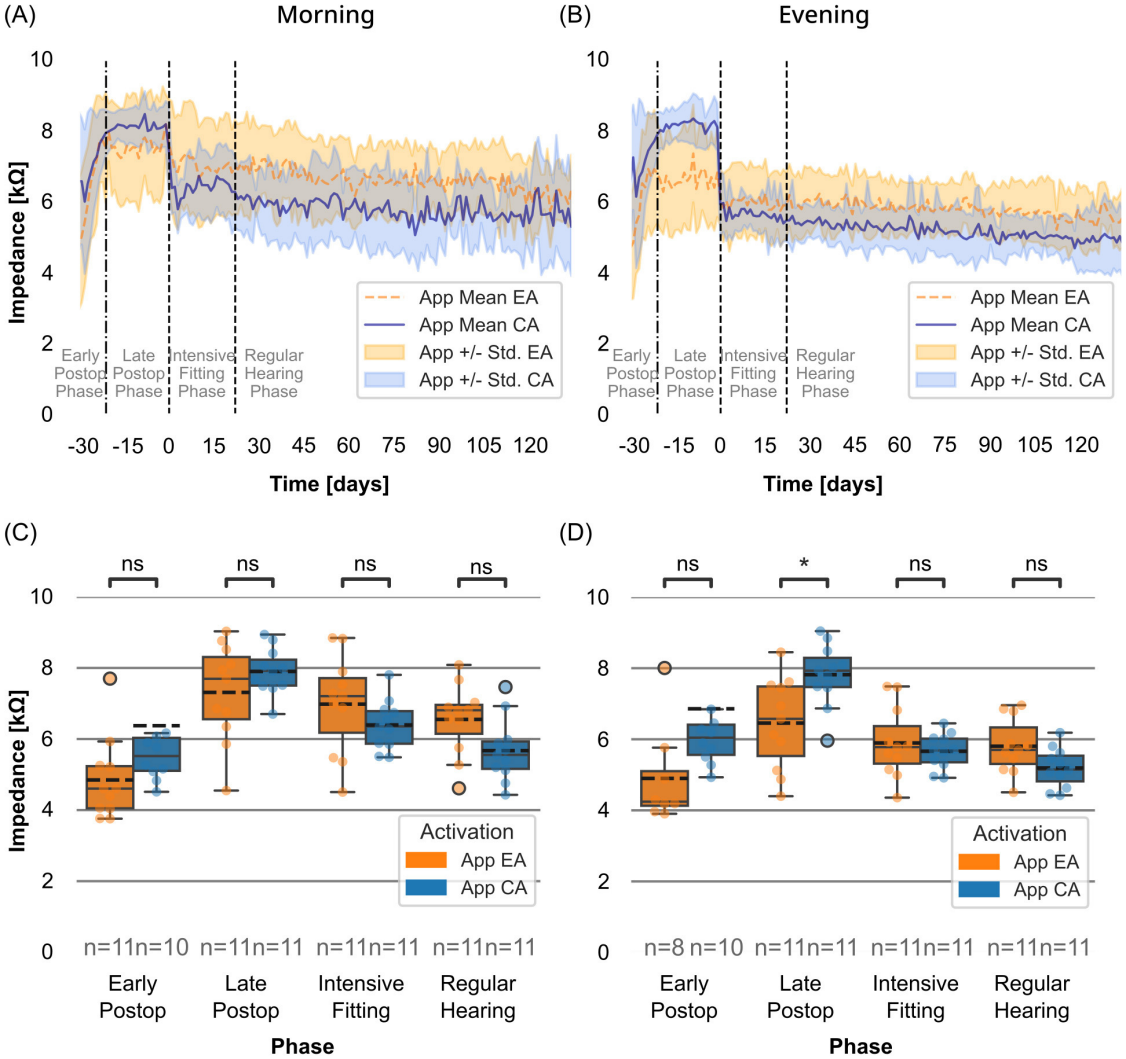
**(A, B)** Daily mean impedance (kΩ) measured with the Telemetry App in the morning **(A)** and evening **(B)** for EA group (dotted orange line) and CA group (solid blue line). Shaded areas represent the standard deviation. Vertical lines mark the postoperative phases. The period 30–22 days before t_0_ includes mixed data from both the early and late postoperative phases. **(C, D)** Distribution of impedance values for the EA and CA groups measured in the morning **(C)** and evening **(D)**, stratified by postoperative phases. Boxes show interquartile ranges with medians and means (dashed line); whiskers depict min/max values. T-test with Bonferroni correction (*0.01 <*p* ≤ 0.05; ns: *p*> 0.05).

The means and standard deviations of the impedance values over time averaged across all channels (C1–C12) are presented in Figure 3, shown separately for the EA (orange, *n* = 11) and CA (blue, *n* = 11) groups. The data are also shown separately for morning (Figure 3A) and evening (Figure 3B) measurements. As mentioned above, the start of the intensive fitting week was used as the reference timepoint (*t*_0_).

Individual measurements for each participant, averaged across all channels, are shown in Supplementary Figure S2. Note that in these figures, the day of implantation was used as the reference point (*t*_0_).

#### Morning impedance measurements in the app cohort

In the early postoperative phase, the two activation groups exhibited comparable values. During the late postoperative phase, both activation groups showed a trend toward higher values in mean morning impedance compared to mean evening impedance (Figure 3A). The EA group showed slightly lower mean impedance compared to the CA group during this phase. However, both groups reached comparable maximum values prior to the start of the intensive fitting week (EA: 8.17 ± 0.84 kΩ, 1 day before the start of the intensive fitting week (*n* = 8); CA: 8.46 ± 0.64 kΩ, 8 days before the start of the intensive fitting week (*n* = 6)).

During the intensive fitting phase, the start of the intensive fitting week was not associated with significant impedance changes for the EA group, although slightly lower impedance (<0.5 kΩ lower) was observed at the last day (*t* = 4) of the intensive fitting week. In contrast, the CA group showed a more pronounced decrease in impedance (>1 kΩ) between the first and last day of the intensive fitting week. As a result, the mean morning impedance of the CA group was lower than that of the EA group at the end of the intensive fitting phase, but with a mean difference of <1 kΩ. Despite this, both groups' values converged to ~6 kΩ during the regular hearing phase.

Figure 3C shows the distributions of all usable impedance values for morning measurements, stratified by activation group. Within each postoperative phase, no significant differences in mean morning impedance values were found between the EA and CA groups (Table 1).

**Table 1:** Statistical analysis of the differences between the early activation (EA) and conventional activation (CA) groups at each postoperative phase.

**Phase**	**EA (kΩ)**	**CA (kΩ)**	***p*-Value**
Early postoperative	4.85	6.38	0.18 (MWU), 0.67
Late postoperative	7.32	7.90	0.87
Intensive fitting	6.98	6.40	0.92
Regular hearing	6.55	5.67	0.17

The mean morning impedance values are shown. Note that *n*= 10 for the CA group in the early postoperative phase, *n*= 11 for all other conditions. The p-values shown are for t-tests, except for the early postoperative phase, where the Mann–Whitney U (MWU) p-value is also shown.

Standardized values corresponding to Tables 1, 2 are provided in Supplementary Tables S2, S3. *Z*-scoring was applied to the mean impedance values across the entire cohort to allow for comparability across participants.

**Table 2:** Statistical analysis of the differences between the early activation (EA) and conventional activation (CA) groups at each postoperative phase.

**Phase**	**EA (kΩ)**	**CA (kΩ)**	***p*-Value**
Early postoperative	4.90	6.86	0.06 (MWU), 0.38
Late postoperative	6.46	7.82	0.04
Intensive fitting	5.90	5.66	1.00
Regular hearing	5.80	5.19	0.19

The mean evening impedance values are shown. Note that *n*= 8 for the EA group and *n*= 10 for the CA group in the early postoperative phase, *n*= 11 for all other conditions. The p-values shown are for t-tests, except for the early postoperative phase, where the Mann–Whitney U (MWU) p-value is also shown.

#### Evening impedance measurements in the app cohort

In the EA group, the mean evening impedance values were consistently lower than the mean morning impedance values throughout the entire study period (Figure 3B). Overall, evening impedance values followed a trend similar to the morning values, except in the late postoperative phase, where evening impedance plateaued at ~6.51 ± 1.30 kΩ (*n* = 11).

In the CA group, similar mean morning and evening impedance values were observed across the entire analyzed period, and the same overall trend was observed for both measurement times. However, a notable decrease in evening impedance occurred between 1 day before the start of the intensive fitting week (−1 day: 8.10 kΩ, *n* = 6) and the first day of the intensive fitting week (*t*_0_: 5.93 kΩ, *n* = 9), corresponding when the first electrical stimulation was applied in this group.

Figure 3D shows the distributions of all usable impedance values for evening measurements, stratified by activation group. During the late postoperative phase, impedance values were significantly higher in the CA group compared to the EA group (*p* = 0.04). For the other phases, no significant differences in mean evening impedance values were found between the two groups (Table 2).

### Morning-to-evening differences in app impedance

**Figure 4:**
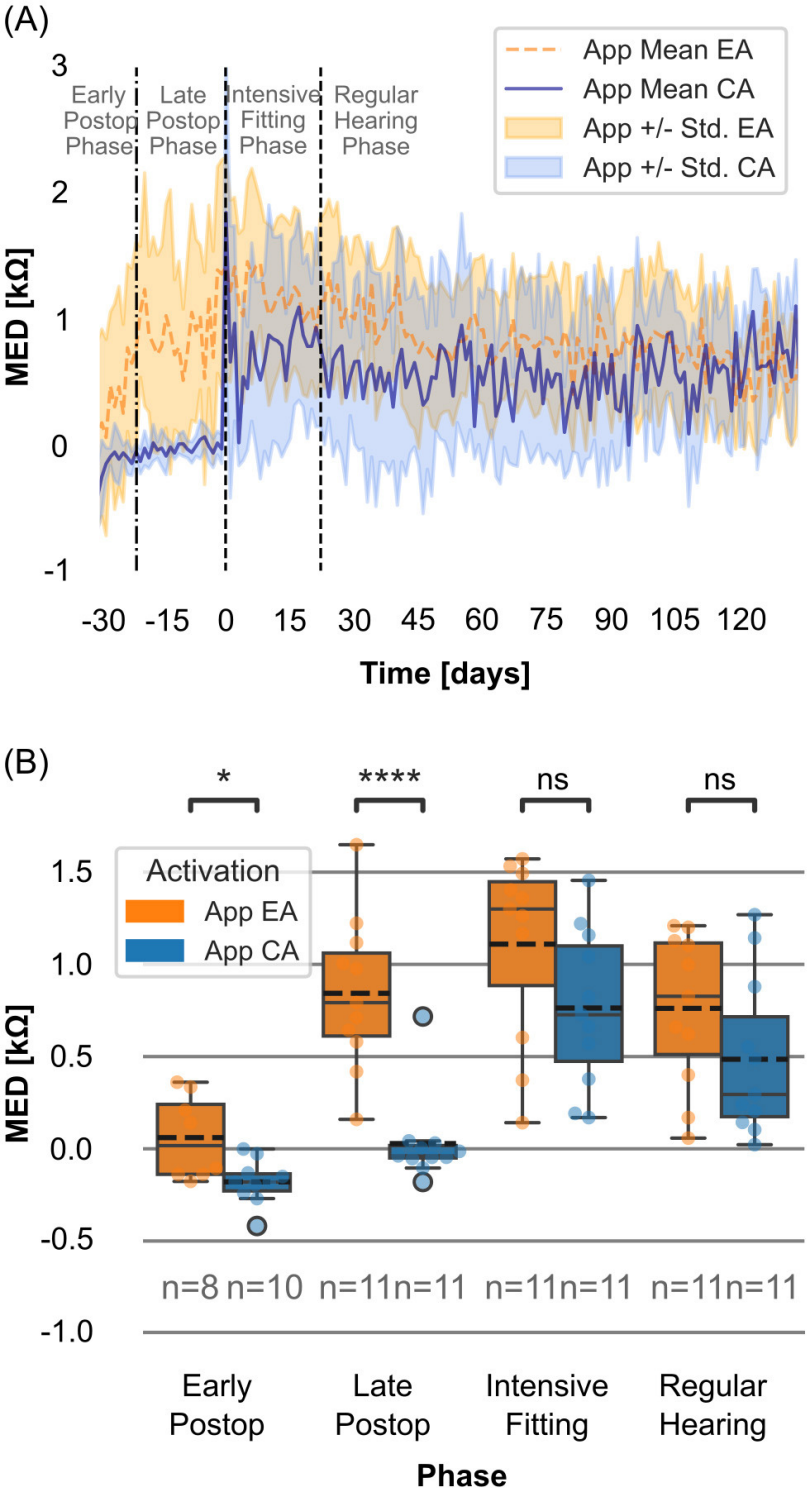
**(A)** Daily mean morning-to-evening differences in impedance (MED; kΩ, morning-evening) measured with the Telemetry App for EA group (dotted orange line) and CA group (solid blue line). Shaded areas representing standard deviation. The reference point (t_0_) is the start of the intensive fitting week. The period 30–22 days before t_0_ includes mixed data from both the early and late postoperative phases. **(B)** Distributions of MED (kΩ) for the EA and CA groups within the postoperative phases. Boxes show interquartile ranges with medians and means (dashed line); whiskers depict min/max values. T-test with Bonferroni correction (****p ≤ 0.0001; *0.01 <*p* ≤ 0.05; ns: *p* > 0.05).

The EA group exhibited a larger mean MED in impedance values during the late postoperative phase (Figure 4A), reaching a maximum value of ~1.4 kΩ 1 day before the start of the intensive fitting week (*n* = 8). In contrast, the CA group exhibited a mean MED close to zero during the late postoperative phase, with a value of ~0.02 kΩ 1 day before the start of the intensive fitting week (*n* = 6).

At the start of the intensive fitting week (*t*_0_), the CA group showed an initial increase in mean MED, followed by a pronounced decrease. During the regular hearing phase, the mean MED of the CA group fluctuated around 0.5 kΩ (*n* = 11).

For the EA group, mean MED in impedance steadily decreased over time, reaching values similar to those of the CA group by the end of the regular hearing phase (day 132: EA: 0.52 kΩ (*n* = 5), CA: 0.54 kΩ (*n* = 4)).

When all usable measurements were compared within each postoperative phase, significant differences were observed in mean MED between the EA and CA groups during the early and late postoperative phases (Table 3, Figure 4B).

**Table 3:** Statistical analysis of the differences between the mean MED values for the early activation (EA) and conventional activation (CA) groups at each postoperative phase.

**Phase**	**EA (kΩ)**	**CA (kΩ)**	***p*-Value**
Early postoperative	0.06	−0.18	0.04
Late postoperative	0.85	0.03	<0.001 (MWU), <0.001
Intensive fitting	1.11	0.76	0.38
Regular hearing	0.76	0.49	0.57

The mean impedance values are shown. Note that n = 8 for the EA group and n = 10 for the CA group in the early postoperative phase, n = 11 for all other conditions. The p-values shown are for t-tests, except for the late postoperative phase, where the Mann–Whitney U (MWU) p-value is also shown.

A comparison of the MED within each postoperative phase clustered by cochlear regions (apical, medial and basal) is shown in Supplementary Figure S3.

### MAESTRO impedance data comparison: app vs. routine cohort

**Figure 5:**
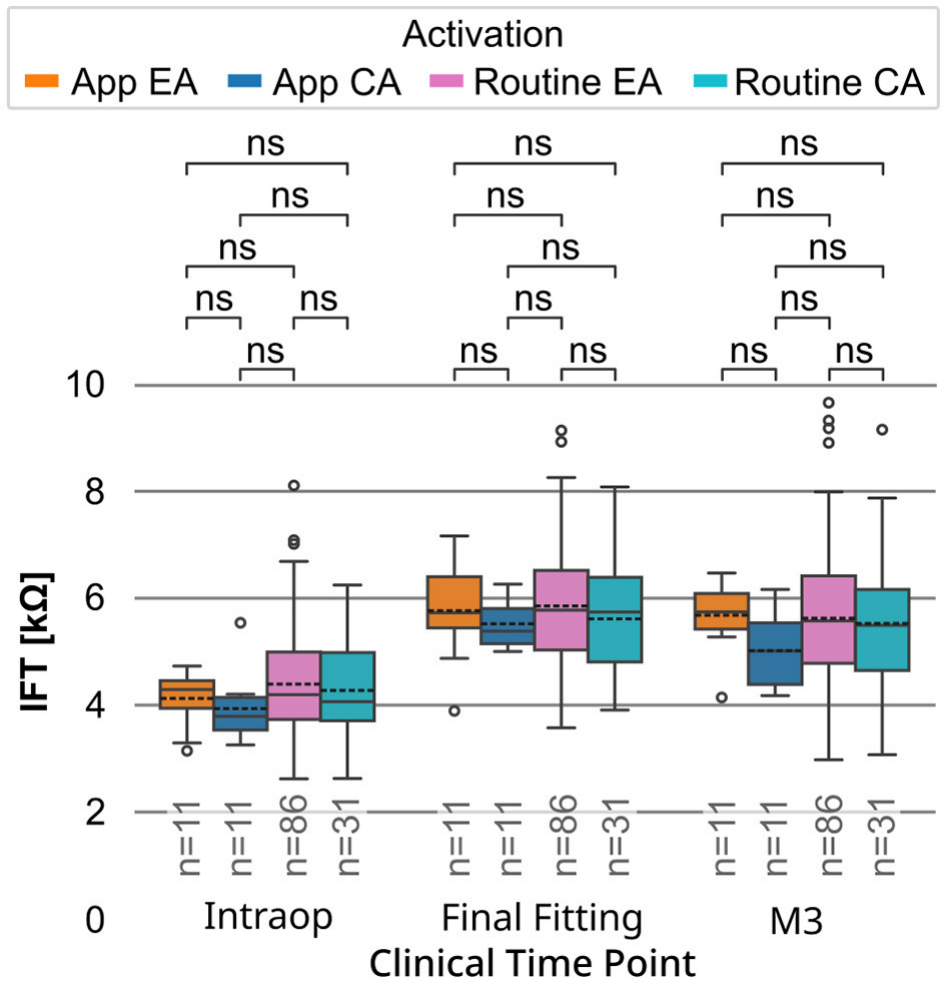
Distributions of IFT values (kΩ) for both activation groups of the app cohort (EA: orange, CA: blue) and a retrospective routine cohort (EA: purple, CA: turquoise) at clinical time points: intraoperative (intraop), end of intensive fitting week (final fitting), and 3-month follow-up (M3). Boxes show interquartile ranges with medians and means (dashed line); whiskers depict min/max values. T-test with Bonferroni correction (ns: *p* > 0.05).

Figure 5 presents the distributions of IFT values for all channels (C1–C12), for both the app cohort and the larger routine cohort from the clinical MAESTRO software, each stratified by their activation strategy (EA or CA). At the defined clinical time points—intraoperative (intraop), end of intensive fitting week (final fitting), and M3—no significant differences in IFT values were observed between the app cohort (EA: *n* = 11, CA: *n* = 11) and the routine cohort (EA: *n* = 86, CA: *n* = 31), regardless of activation strategy. Additionally, within both the app cohort and the routine cohort, the EA and CA subgroups did not differ significantly at any of the clinical time points.

## Discussion

### Usage rate of the Telemetry App

The majority of participants used the Telemetry App daily, as indicated by an overall usage rate of 66% over the 4-month observation period. However, for several individual participants there was incomplete data collection, particularly in the early postoperative phase. The relative sparsity of data collection in this phase may be attributed to multiple factors. Swelling at the implantation site could lead to insufficient magnetic coupling between the audio processor and the implant coil, while postoperative pain might temporarily impede processor use. Additionally, in the first days after surgery, some participants in the EA group may have recorded impedance values without actually using their device continuously. This is due to the routine clinical recommendation that new CI users refrain from wearing the processor until the stitches are removed, typically around 10 days postsurgery.

An analysis of individual participant data suggests that once a participant had completed an impedance measurement using the app, they were likely to continue performing measurements for the duration of the study. Only one participant (Supplementary Figure S2, CA #03) discontinued participation at ~2 months postimplantation. All other participants who had discontinuities resumed their measurements. Conducting continuous impedance measurements twice daily over a period of 4 months may represent a substantial burden for users, which could explain the occasional gaps in the data collection.

Incomplete data collection was also observed in Parreño et al. (27), who collected daily self-measured impedance data over a 30-day period. Given that the present study collected measurements over a substantially longer timeframe, it was expected that some unmeasured timepoints would occur. This factor should be carefully considered when interpreting longitudinal impedance trends, particularly when calculating group means, as peaks in the data may be due to fluctuations in the number of users who performed a measurement at a given timepoint.

To improve measurement reliability in the future, it would be desirable to automate impedance measurements, ensuring that an initial measurement occurs automatically when the processor is first worn. A second measurement could then be triggered after several hours of processor use, eliminating uncertainty about whether users performed both pre and poststimulation impedance measurements.

This uncertainty may explain why some individual morning impedance values were not consistently higher than compared to evening values. For example, one participant (Supplementary Figure S2, CA #02) reported performing morning measurements after breakfast, meaning the processor had already been worn for ~45 min. In such cases, electrical stimulation may have already influenced the impedance values before the morning measurement was taken.

### Individual participant effects

Due to the relatively small cohort size in this study, individual participant effects may have had a strong impact on the results. Factors such as variations in processor wearing time, the number of switch-ons per day, and the level of environmental sound exposure could contribute to impedance variability. Additionally, individual foreign body reactions following CI surgery should be considered (34). Previous research has suggested a link between immunological changes in the cochlea and increased electrode impedance (5, 7). Neuburger et al. (35) further demonstrated a strong temporal association between the increase in impedance levels and inflammatory processes in the cochlea (e.g., labyrinthitis), and even observed a connection between increased impedance levels and the presence of common colds. Regular medication usage, as well as infections and the associated use of medications, may influence daily impedance values.

We also observed potential associations between extreme impedance levels and medical events. One participant experienced severe headaches several days after surgery, coinciding with high impedance values (>15 kΩ, Supplementary Figure S2, CA #07). Another participant recorded impedance values exceeding 20 kΩ in the early postoperative phase and discontinued measurements until the intensive fitting week (Supplementary Figure S2, CA #08). Notably, this participant also postponed the intensive fitting week due to a heart attack, which may have influenced the observed data. Lower impedance values and less pronounced MEDs were observed in one participant who regularly used an asthma spray containing steroids (Supplementary Figure S2, EA #02). Steroid administration is known to influence impedance values (21–23). To better understand the impact of steroid-containing asthma sprays on CI impedance, further studies with a larger cohort are necessary. These observations suggest that impedance values are not only strongly correlated with inner ear physiology, but may also be influenced by systemic conditions or patient-specific physiological factors unrelated to the inner ear.

### Overall trends in app impedance

The temporal trends in mean evening impedance recorded via the Telemetry App were consistent with expected patterns. In the CA group, a gradual increase in impedance was observed until the onset of electrical stimulation (Figures 3B, D) (36). The EA group followed a similar pattern initially, but impedance values plateaued at a lower level during the late postoperative phase. This difference resulted in significantly lower impedance values in the EA group just before the intensive fitting week, a finding consistent with multiple reports in the literature (30, 37, 38). In the CA group, the decrease in impedance following the onset of electrical stimulation also aligns well with the results of previous studies with conventionally activated implants (11, 36, 39, 40). Previous studies have also observed that after initial activation, impedance values between EA and CA groups tend to converge at follow-up visits (30, 37, 38).

In contrast, a recent study reported significantly lower impedance levels with EA compared to CA at up to 2 years postimplantation (41). The reported difference in median impedance between the groups was 0.35 kΩ at 1 year and 0.43 kΩ at 2 years. Although statistically significant, the magnitude of this effect is quite small. Assuming that impedance differences <1 kΩ are not clinically relevant (37), the findings from present study and those reported previously (41) indicate that after the initiation of electrical stimulation, no clinically relevant differences exist between EA and CA fitting strategies in terms of mean impedance values.

### Impedance measured with the app vs. with the MAESTRO IFT

The impedance results obtained via the Telemetry App suggest a tendency toward lower mean impedance values in the CA group following the onset of electrical stimulation (Figures 3A, B). Comparing the IFT values collected at routine follow-up appointments revealed the same trend (Figure 5), but the difference was not statistically significant. In addition, when the IFT values of the larger routine cohort were compared, the same trend was again observed, but the differences were again not statistically significant (Figure 5).

For the EA fitting approach, the mean impedance was 5.77 kΩ (*n* = 11) for the app cohort and 5.86 kΩ (*n* = 86) for the routine cohort at the final fitting appointment. For the CA fitting approach, the mean impedance was 5.52 kΩ (*n* = 11) for the app cohort, and 5.61 kΩ (*n* = 31) for the routine cohort at the final fitting appointment. These mean values are comparable to those previously reported in clinical studies. For example, Prenzler et al. (36) reported data from a control group of MED-EL FLEX28 users who received their first electrical stimulation at the start of the intensive fitting week, similar to the CA group in this study. The mean impedance across all electrodes was ~5.5 kΩ (*n* = 5) a few days after the first electrical stimulation during the fitting week.

### Morning-to-evening differences in app impedance

In general, impedance values were higher in the morning than in the evening, a pattern consistent with the findings of Mushtaq et al. (32). This is likely explained by the absence of electrical stimulation overnight. A recent literature review described the observation of a decrease in impedance possibly as result of cell detachment triggered by electrical stimulation (42).

By comparing the EA and CA groups, we observed that the onset of daily impedance fluctuations was linked to the onset of electrical stimulation. For the EA group, the daily fluctuation began in the late postoperative phase, whereas for the CA group, there was effectively no MED until the intensive fitting phase (Figure 4). Notably, in the EA group, mean MED in impedance exceeding 1 kΩ (Figure 4B) were observed throughout the late postoperative, intensive fitting and regular hearing phase. The comparison MED across different cochlear regions (apical, medial, and basal) between EA and CA groups reveals a pattern similar to that observed when averaging across all contacts (Supplementary Figure S3). The MED is most pronounced in the apical region (C1–C5), which may, however, be influenced by the single-sided contacts of the electrode array. Whether these findings have a clinically relevant impact, such as on hearing performance, should be investigated in more detail in a subsequent study.

Impedance fluctuations exceeding 1 kΩ are particularly relevant during the onset of electrical stimulation, as they affect the initial fitting parameters and may compromise the delivery of adequate current for optimal auditory perception (19). The data indicate that impedance fluctuations were already present in some participants in the EA group during the late postoperative phase. As discussed earlier, most did not actively use their processor throughout the day during the first 10 days postoperative, likely only wearing it briefly for app measurements. This raises the question of whether MED in impedance exceeding 1 kΩ may also occur during the early postoperative phase with the EA strategy. However, this early phase in the EA group should be interpreted with caution, as inconsistent processor use may have limited the actual amount of electrical stimulation received. This represents a limitation of the current dataset and should be addressed more specifically in future studies.

Since some parameters of fitting maps depend on impedance values, and considering that initial activation in the EA group often occurs during the early postoperative phase, the potential implications of fluctuating impedances on fitting adjustments could be considered in future clinical practice. Specifically, as impedance influences the relationship between supply voltage and current, elevated impedance levels can limit the maximum deliverable current due to device voltage constraints. In cases where higher charge levels are required for effective stimulation, clinicians may need to compensate by adjusting pulse width. Therefore, awareness of impedance fluctuations—such as consistently higher values in the morning—may be particularly relevant during the initiation of electrical stimulation, especially in certain patients.

For the CA group, significantly higher impedance values compared to the EA group—both morning and evening—were observed prior to the onset of electrical stimulation (Figures 3C, D). As for the EA group, these elevated preactivation impedance values may result in insufficient initial electrical current for optimal auditory stimulation. Furthermore, it is important to note that in the present study, averaging across all channels decreased the overall effect, meaning impedance fluctuation may be significantly higher on individual channels than the averaged data suggests. While the direct connection between impedance fluctuations and fitting adjustments or speech perception has not been conclusively established, it is possible that recalibration and adjustments in response to elevated impedance values could contribute to improved sound perception. At the German Hearing Center, a fitting week is commonly practiced, during which intensive fitting is conducted over 5 days, and the factor of impedance fluctuations may be addressed.

### Implications for current and future CI care

Based on these findings, when conducting impedance measurements with the clinical software method (IFT), a second impedance measurement after several hours of processor use could be considered in future studies or clinical protocols to capture more stable impedance values after an initial fitting session. Alternatively, incorporating a second fitting day can ensure several hours of electrical stimulation between impedance measurements. This approach would allow for more clinically relevant impedance data to be obtained.

To account for the daily fluctuations in impedance levels, comparisons between Telemetry App measurements and routine IFT measurements should use only the evening app measurements, as both are collected after a period of electrical stimulation. In this study, the mean evening impedance measured with the Telemetry App during the intensive fitting phase of the CA group (5.66 kΩ, *n* = 11, Figure 3D) aligns well with the data from the above-mentioned literature.

The implementation of automated, rather than manual, app impedance measurements could provide more reliable data by ensuring impedance values are recorded at consistent time points, particularly after several hours of electrical stimulation. These measurements could yield a more precise input for fitting adjustments.

With daily measurements, the ability to detect longitudinal impedance trends at high temporal resolution could facilitate the early detection of abnormalities, enabling timely clinical intervention. For instance, given the strong temporal association between increased impedance values and inflammatory processes such as labyrinthitis (35), remote monitoring could facilitate the early identification of inflammatory episodes, leading to prompt pharmacological intervention which could potentially prevent further complications. In addition, continuous impedance monitoring may be able to leverage the value provided by conventional impedance telemetry, such as supporting the detection of electrode malfunction and biological changes like tissue growth. A higher measurement frequency could facilitate earlier detection of such events. It may also be used to supplement remote fitting procedures (20, 43, 44). These applications could significantly enhance individualized and decentralized care in CI users, particularly in rural or underserved areas. Early research suggests that matrix-based impedance measurements may be used to estimate intracochlear positioning of the electrode array (45–47). The current version of the Telemetry App performs only clinical impedance measurements, but it is not technically infeasible to perform full-matrix impedance measurements remotely. If this functionality were to be added, daily monitoring could also facilitate early detection of changes in array positioning.

Moreover, with the healthcare system facing increasing demands due to the growing number of CI recipients, remote monitoring solutions could help reduce the burden on clinical resources by reducing the frequency of routine in-person checkups. Tools like the app evaluated in this study may help to facilitate a shift toward event-triggered and patient-specific follow-up care.

Finally, beyond its direct clinical applications, this study reinforces the broader importance of patient empowerment and user engagement in CI rehabilitation. By actively engaging in daily impedance monitoring, users may develop a greater sense of involvement in their care, which can contribute to a stronger feeling of control over their hearing health. While impedance data alone does not directly reflect device performance, additional app features, such as hearing tests or device diagnostics, could provide users with more concrete insights into their device's status and performance.

## Conclusion

The Telemetry App enabled remote impedance measurements in cochlear implant users. With a higher measurement frequency to those typically performed during clinical routine visits, this approach allowed for detailed characterization of impedance changes throughout different postoperative phases up to 4 months postoperatively, particularly in relation to the onset of electrical stimulation. No clinically relevant difference was observed between the EA and CA groups in terms of mean impedance values. Daily impedance fluctuations were evident once electrical stimulation was introduced, with consistently lower evening impedance values.

## Data Availability

The raw data supporting the conclusions of this article will be made available by the authors, without undue reservation.
