# Supplementary material for: App-based daily self-measurement of impedance in cochlear implant users
Source: Front Neurol. 2025 Jul 4;16:1618031. doi: 10.3389/fneur.2025.1618031 (PMC12273450; doi:10.3389/fneur.2025.1618031)
Supplement: Supplementary file 1 [file Data_Sheet_1.pdf]

Supplementary Material

App-based daily self-measurement of impedance in cochlear implant users

Sarah Vormelcher[1,2*], Cornelia Batsoulis[2], Daniel Kley[1], Michael Mair[3], Andreas Büchner[4]

[1] Department of Otorhinolaryngology, Hannover Medical School, Hannover, Germany.

[2] MED-EL Research Center, MED-EL Medical Electronics GmbH, Hannover, Germany.

[3] MED-EL R&D, MED-EL Medical Electronics GmbH, MED-EL Headquarters, Innsbruck, Austria.

[4] Department of Otorhinolaryngology and Cluster of Excellence "Hearing4all", Hannover Medical School, Hannover, Germany.

*** Correspondence:**
Sarah Vormelcher
vormelcher.sarah@mh-hannover.de

Supplementary Figures

Material and Methods

Study Design, Inclusion Criteria, and Participant Details

Supplementary Table S1*: Demographic data of the n=28 Telemetry App participants for the two activation groups: early activation (EA) and direct activation (DA). ID (#) – Identifier assigned to participants with > 33% app usage rate, used for individual impedance representations (see Supplementary Figure S2).*

No.	Gender	Age at Implantation	Side of Implantation	Activation Group
01	Male	43.4	right	EA #01
02	Male	70.3	right	EA #02
03	Male	72.0	right	EA #03
04	Male	78.9	left	EA #04
05	Male	65.9	right	EA #05
06	Female	36.9	right	EA #06
07	Male	63.7	right	EA #07
08	Male	70.3	right	EA #08
09	Female	59.5	right	EA #09
10	Male	53.6	left	EA #10

11	Female	58.7	left	EA #11
12	Female	53.4	left	EA
13	Male	30.9	left	EA
14	Male	72.7	right	CA #01
15	Male	67.8	right	CA #02
16	Male	66.7	right	CA #03
17	Male	38.6	left	CA #04
18	Female	49.1	left	CA #05
19	Male	59.7	right	CA #06
20	Female	55.4	left	CA #07
21	Male	50.6	right	CA #08
22	Male	81.0	right	CA #09
23	Male	78.3	left	CA #10
24	Male	56.5	right	CA #11
25	Male	83.1	right	CA
26	Male	61.1	right	CA
27	Male	32.3	left	CA
28	Female	21.8	left	CA

Results

Usage Rate of the Telemetry App

Supplementary Figure S1 displays histograms illustrating the temporal distribution of measurement time points categorized as morning and evening. A total of 96.7% of morning measurements occurred before 12:00 PM, while 97.7% of evening measurements were conducted after 4:00 PM.

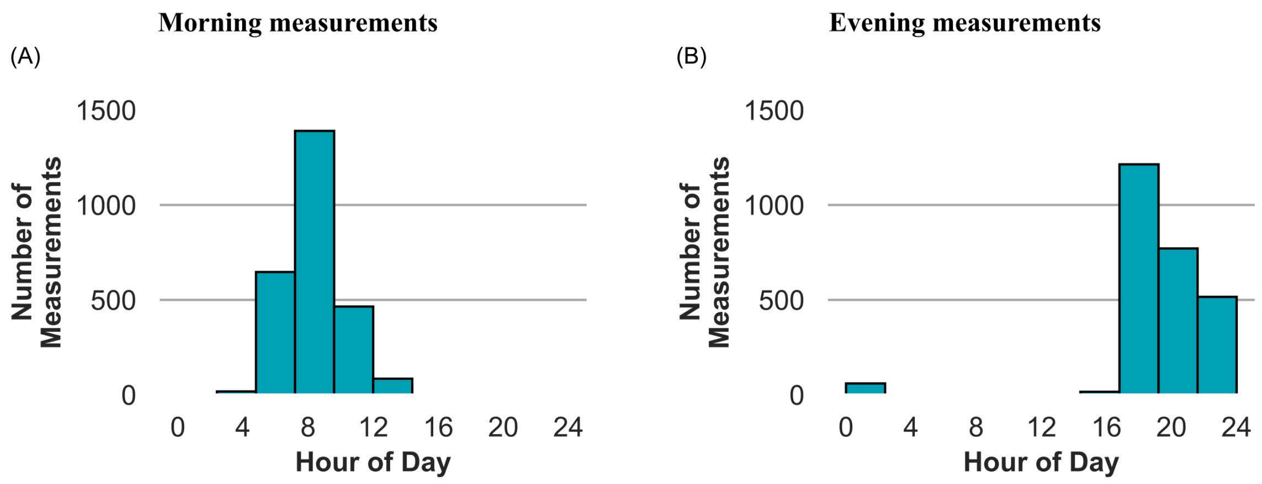

Supplementary Figure S1*: Histogram of app-based impedance measurements for the 22 analyzed participants, showing the distribution of measurements by hour of the day during the period between*

clinic appointments (postoperative implant check to M3). (A) Morning measurements, (B) Evening measurements.

Impedance Measurements across time in the App Cohort

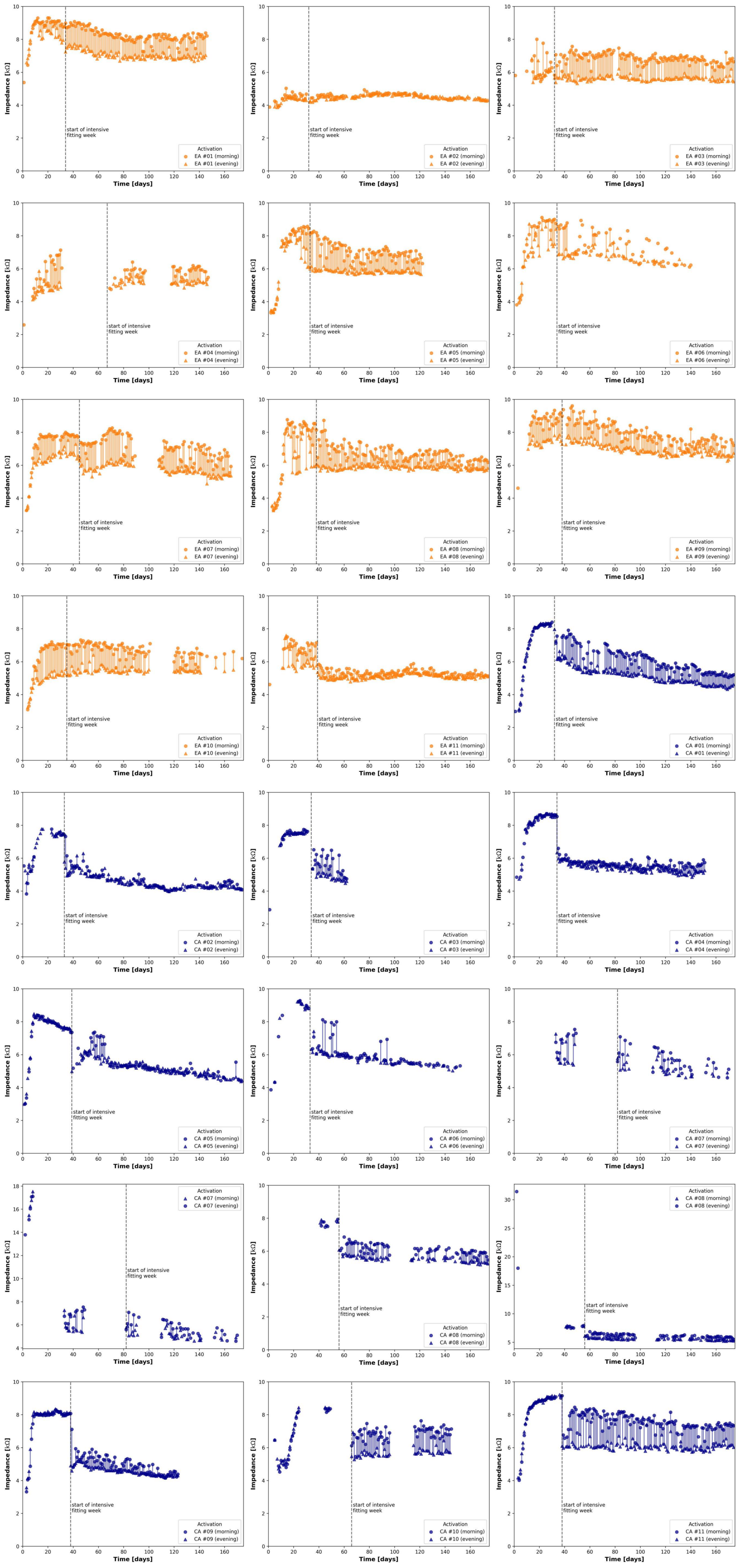

Impedance [kΩ]
Time [days]
start of intensive fitting week
Activation
EA #01 (morning)
EA #01 (evening)
EA #02 (morning)
EA #02 (evening)
EA #03 (morning)
EA #03 (evening)
EA #04 (morning)
EA #04 (evening)
EA #05 (morning)
EA #05 (evening)
EA #06 (morning)
EA #06 (evening)
EA #07 (morning)
EA #07 (evening)
EA #08 (morning)
EA #08 (evening)
EA #09 (morning)
EA #09 (evening)
EA #10 (morning)
EA #10 (evening)
EA #11 (morning)
EA #11 (evening)
CA #01 (morning)
CA #01 (evening)
CA #02 (morning)
CA #02 (evening)
CA #03 (morning)
CA #03 (evening)
CA #04 (morning)
CA #04 (evening)
CA #05 (morning)
CA #05 (evening)
CA #06 (morning)
CA #06 (evening)
CA #07 (morning)
CA #07 (evening)
CA #07 (morning)
CA #07 (evening)
CA #08 (morning)
CA #08 (evening)
CA #08 (morning)
CA #08 (evening)
CA #09 (morning)
CA #09 (evening)
CA #10 (morning)
CA #10 (evening)
CA #11 (morning)
CA #11 (evening)

Supplementary Figure S2*: Daily impedance values (kΩ) measured with the Telemetry App for participants with > 33% app usage rate. The day of implantation (t_0) serves as the reference point. Data from the EA group (orange) and CA group (blue) are shown, with morning measurements indicated by dots and evening measurements by triangles. Vertical lines mark the individual start of the intensive fitting week. Note that for two participants in the CA group (CA #07, CA #08), an additional plot with an extended vertical axis is provided.*

-Morning impedance measurements in the app cohort

Supplementary Table S2*: Statistical analysis of the differences between the early activation (EA) and conventional activation (CA) groups at each postoperative phase. The z-scores of the mean morning impedance values are shown. Note that n = 10 for the CA group in the early postoperative phase, n = 11 for all other conditions. The p-values shown are for t-tests, except for the early postoperative phase, where the Mann-Whitney U (MWU) p-value is also shown.*

**Phase**	**EA**	**CA**	***p*-value**
Early Postoperative	-0.31	0.08	0.18 (MWU), 0.56
Late Postoperative	-0.27	0.24	0.85
Intensive Fitting	0.22	-0.27	0.90
Regular hearing	0.26	-0.53	0.17

-Evening impedance measurements in the app cohort

Supplementary Table S3: *Statistical analysis of the differences between the early activation (EA) and conventional activation (CA) groups at each postoperative phase. The z-scores of the mean evening impedance values are shown. Note that n = 8 for the EA group and n = 10 for the CA group in the early postoperative phase. n = 11 for all other conditions. The p-values shown are for t-tests, except for the early postoperative phase, where the Mann-Whitney U (MWU) p-value is also shown.*

**Phase**	**EA**	**CA**	***p*-value**
Early Postoperative	-0.34	0.41	0.06 (MWU), 0.46
Late Postoperative	-0.49	0.53	0.04
Intensive Fitting	0.12	-0.17	1.00
Regular hearing	0.36	-0.43	0.19

Morning-to-Evening Differences in App Impedance

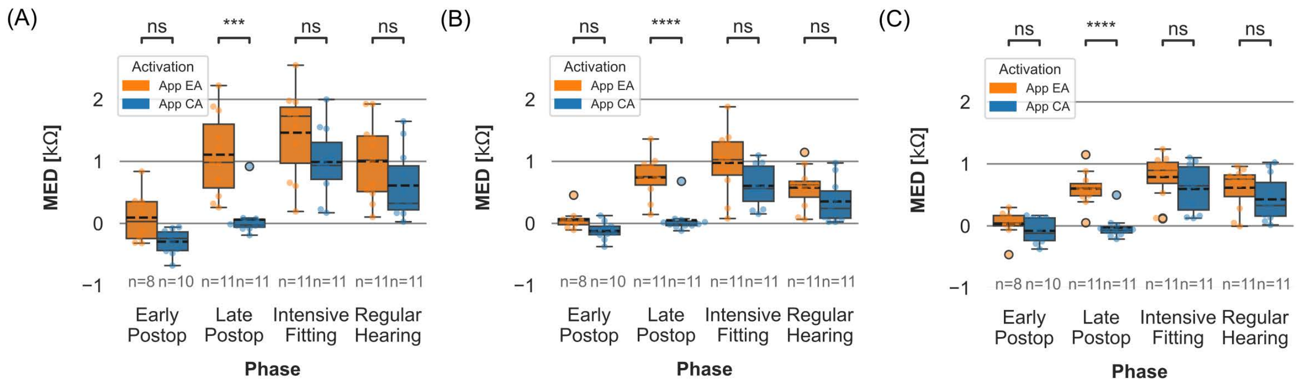

Supplementary Figure S3: *Distributions of MED (kΩ) for the EA and CA groups within the postoperative phases for the different cochlear regions. (A) Apical: electrode contact 1-5, (B) Medial: electrode contact 6-9 and (C) Basal: electrode contact 10-12. Boxes show interquartile ranges with medians and means (dashed line); whiskers depict min/max values. T-test with Bonferroni correction (****: $p \leq 0.0001$; ***: $0.0001 < p \leq 0.001$; ns: $p > 0.05$).*
